# Ceramic Biomaterial Pores Stereology Analysis by the Use of Microtomography

**DOI:** 10.3390/ma14092207

**Published:** 2021-04-25

**Authors:** Żaneta Garczyk, Zbigniew Jaegermann, Piotr Duda, Andrzej S. Swinarew, Sebastian Stach

**Affiliations:** 1 Institute of Biomedical Engineering, Faculty of Science and Technology, University of Silesia in Katowice, Będzińska 39, 41-205 Sosnowiec, Poland; piotr.duda@us.edu.pl (P.D.); andrzej.swinarew@us.edu.pl (A.S.S.); sebastian.stach@us.edu.pl (S.S.); 2 Łukasiewicz Research Network—Institute of Ceramics and Building Materials, Cementowa 8, 31-983 Kraków, Poland; z.jaegermann@icimb.pl; 3 Institute of Sport Science, Department of Swimming and Water Rescue, The Jerzy Kukuczka Academy of Physical Education, 40-065 Katowice, Poland

**Keywords:** porous bioceramics, alumina, microtomography apparatus, image processing

## Abstract

The main aim of this study was to analyze microtomographic data to determine the geometric dimensions of a ceramic porous material’s internal structure. Samples of a porous corundum biomaterial were the research material. The samples were prepared by chemical foaming and were measured using an X-ray scanner. In the next stage, 3D images of the samples were generated and analyzed using Thermo Scientific Avizo software. The analysis enabled the isolation of individual pores. Then, the parameters characterizing the pore geometry and the porosity of the samples were calculated. The last part of the research consisted of verifying the developed method by comparing the obtained results with the parameters obtained from the microscopic examinations of the biomaterial. The comparison of the results confirmed the correctness of the developed method. The developed methodology can be used to analyze biomaterial samples to assess the geometric dimensions of biomaterial pores.

## 1. Introduction

Porous materials are characterized by the presence of voids (pores) of various sizes and shapes in a solid material, connected to each other and forming an extensive, irregular mesh. Material porosity is a property of solids that determines the size and number of pores inside the material and describes their distribution in the analyzed area. Porosity is defined as the ratio of the volume occupied by the pores to the total volume of the material and is given as a percentage. It depends on the synthesis parameters as well as chemical structure materials containing open or closed pores. The open pores are interconnected as well as connected to the material surface. The closed pores are isolated, not connected with each other [1,2,3,4]. Closed pores do not take part in biochemical and cytological interactions in the body, and they do not overgrow with connective tissue; they only reduce the actual density of the material. They also do not increase the active surface of the material.

Porous bioceramics are of great importance in medicine [2,3]. The porous surface of implants affects the osseointegration process [1,5,6,7]. Furthermore, porous biomaterials are drug carriers because they allow for the placement of a drug substance in the material pores and then its introduction directly into the human body during implantation. The implant with the drug prevents the formation of inflammation and helps avoid postoperative complications, which improves the patient’s comfort by reducing the treatment time [8,9,10,11].

The porosity of biomaterials is measured using experimental methods, microscopic methods, and X-ray computed microtomography methods.

One of the experimental ways of determining material porosity is the gas expansion or compression method. The gas compression method uses gas (nitrogen, carbon dioxide, argon, or krypton) that penetrates the material open pores. The sample is placed in a gas atmosphere in an airtight vessel. Next, the pressure of the gas is gradually increased to a specific value. The procedure is also repeated for an empty vessel. It allows for designating the sample porosity by determining the volume occupied by its skeleton and the sample volume [12].

Another experimental way of testing porosity is the mercury porosimetry method. The sample is placed in a tank. Then, the air from the chamber is removed using a vacuum pump, and mercury is added. The mercury does not penetrate inside the sample due to surface tension. It allows for determining the total volume of the sample. Then, the pressure is increased, which allows the mercury to penetrate the material pores. The pore size distribution and material porosity can be designated by measuring the increase in mercury pressure and volume. The disadvantage of the method is the destruction of the material as the mercury remains in the sample, and a high mercury pressure may entail sample distortion [12,13,14].

Porous biomaterials must meet the requirements for open and total porosity. However, in addition to determining the porosity, it is also important to characterize the geometric dimensions of pores. The dimensions and types of pores determine the connection of the implant with the tissue by its growing into the biomaterial pores. Moreover, parameter control enables direct control of the drug amount that can be put into the biomaterial pores and delivered to the body.

Experimental methods make it possible to determine the material porosity but not to characterize the geometric dimensions of pores. Microscopic methods allow for the analysis of pore sizes and geometry by observing material cross-sections. Optical or electron microscopy is often used in the analysis of porosity. Optical microscopes are a source of information about 2D objects, but they do not allow for 3D observations [14].

Material porosity is also measured using microtomography. Microtomography uses projections produced from different directions to create cross-sectional and spatial images. The main advantage of computed microtomography is the ability to visualize the internal structure of porous materials at a very high level of detail. This makes it possible to determine many parameters characterizing the geometric dimensions of pores. Moreover, the method is minimally invasive and does not damage the material during testing [15,16].

Despite technological advances, 3D imaging techniques such as X-ray microtomography are often used only for qualitative evaluation of materials through their visualization. Methods for quantifying the internal structure of porous materials are still few and related to specific materials and their applications [17,18,19,20,21,22].

This present research aims to stereologically analyze a porous ceramic biomaterial using micro-computed tomography and characterize the pores of the biomaterial to determine their geometric dimensions.

## 2. Materials and Methods

### 2.1. The Test Material

**Figure 1:**
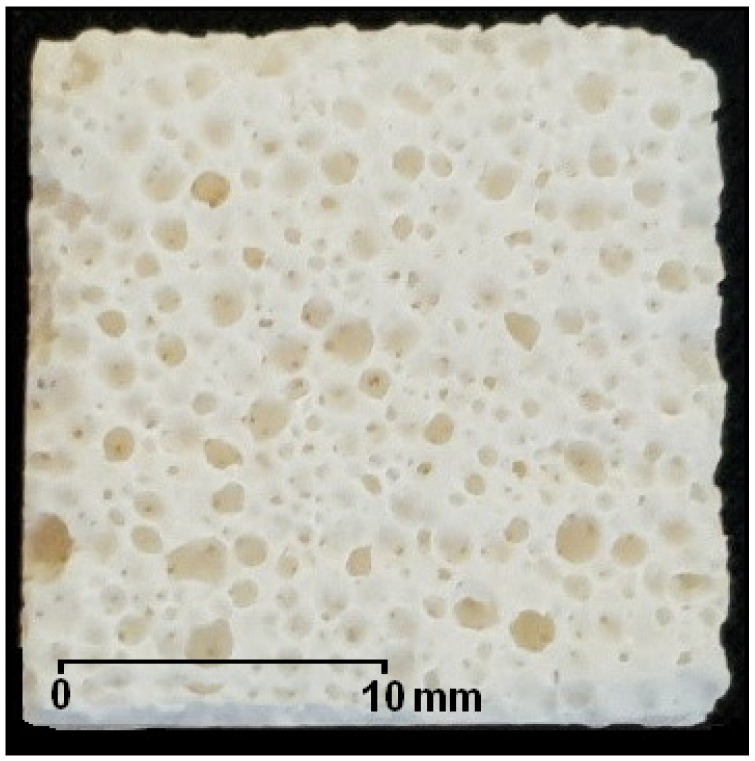
Sample of a porous corundum biomaterial.

Samples of a porous corundum biomaterial were studied (Figure 1). They were prepared by chemical foaming, which involved their molding from a slurry containing fine-grained alumina (61.7% by weight), an aqueous solution of an aluminum oxychloride-based polymer (35.9% by weight), magnesium oxide (1.3% by weight), and calcium carbonate (1.2% by weight) [23]. The starting ingredients were mixed. The calcium carbonate decomposed under the influence of the polymer solution acid reaction, and the emitted CO_2_ foamed the slurry. The gelation of the polymer by neutralizing the reaction of the slurry with the calcium oxide and magnesium oxide allowed the porous biomaterial structure to be preserved. Then, the semi-finished products were fired at 1730 °C.

**Figure 2:**
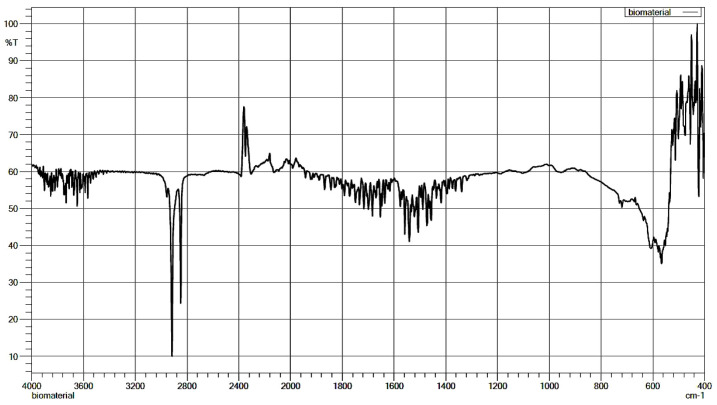
FT–IR ATR analysis of porous corundum biomaterial.

The final formation of the material was approved by FT-IR ATR spectroscopy (Figure 2). Additionally, the analysis revealed residues from the synthesis process in the form of calcium stearate (2918, 2850 cm^−1^) with a high residual carbon dioxide (2342 cm^−1^) content.

The samples, sized 22 mm × 22 mm × 12 mm, were cut from different semi-finished products by the use of a microtome.

### 2.2. Analysis of Microtomographic Images

The X–ray GE Phoenix v|tome|x scanner (Waygate Technologies, Hürth, Germany) was used as the research tool. The microtomographic examination was carried out for 3 samples. The samples were scanned with the same tube parameters (80 μA, 160 kV), providing the same image resolution (13 μm was the voxel size). The duration of a single projection was 150 ms, and the projection consisted of 3 exposures.

**Figure 3:**
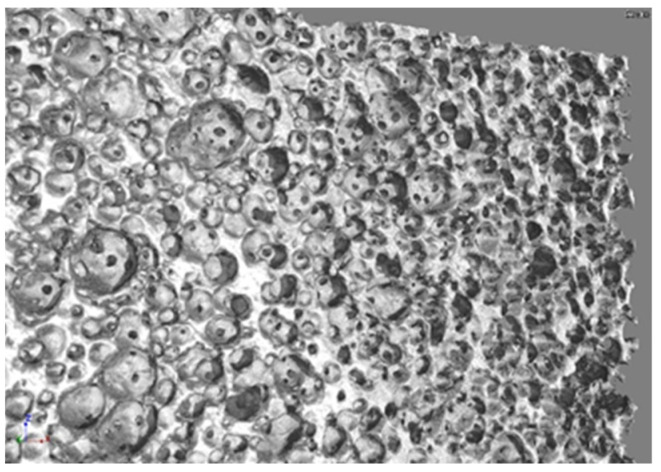
Microtomography image of corundum sample showing the porous surface of the biomaterial.

The 3D images of the samples were generated (Figure 3) from a collection of high-resolution images, which were reconstructed from a series of 2300 X-ray images obtained directly from the microtomography examination.

**Figure 4:**
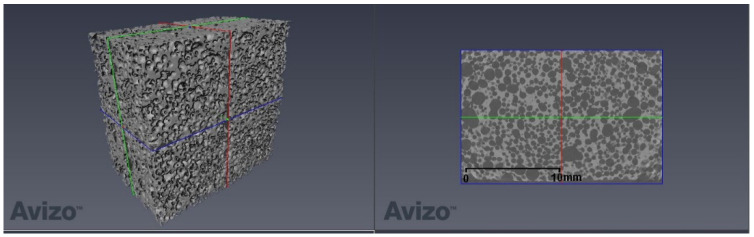
Measurement data for one of the samples—a 3D view and selected layer in the xy plane.

**Figure 5:**
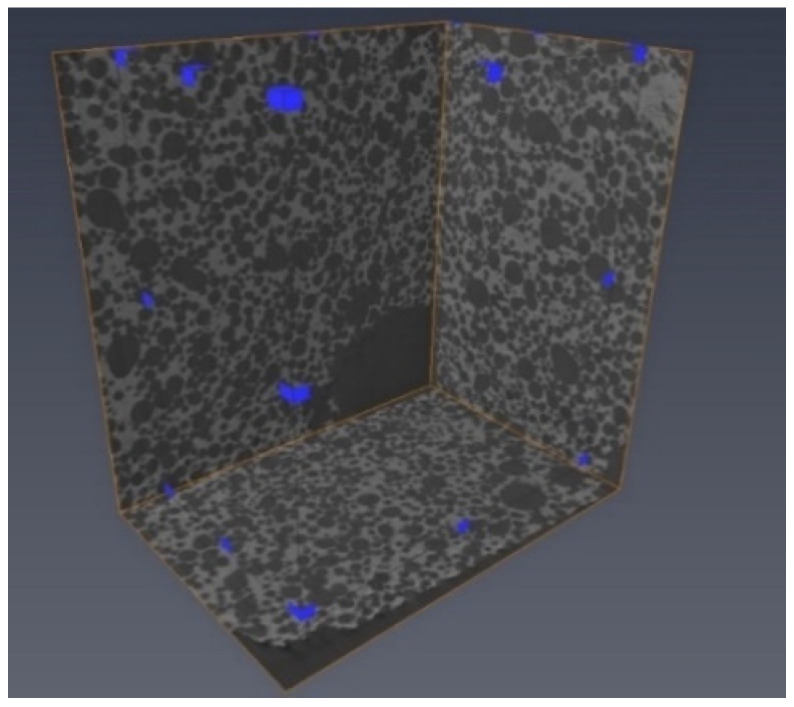
Area of analysis for one of the porous corundum biomaterial samples.

The measurement data were analyzed using the Thermo Scientific Avizo program (version 2019.3, ThermoFisher Scientific, Waltham, MA, USA). The first stage of analysis involved data loading and displaying (Figure 4). Subsequently, the area to be analyzed was separated (Figure 5). The results of operations used in the individual methods are presented for one selected layer.

**Figure 6:**
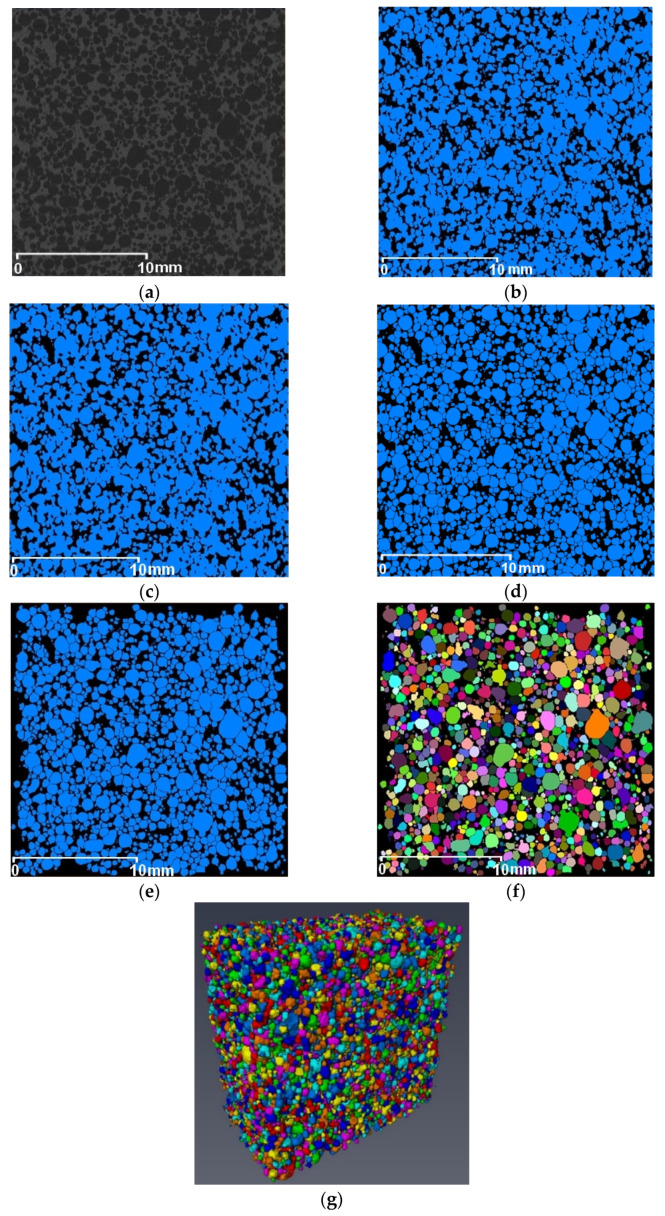
Microtomographic data analysis presented for one of the layers: (**a**) median filter; (**b**) binarization; (**c**) morphological opening; (**d**) disconnection of interconnected pores; (**e**) deletion of pores cut with the image frame; (**f**) result of the analysis; (**g**) result of the analysis—3D view.

A median filter was then used (interpretation: 3D, type: iterative, number of iterations: 3, neighborhood: 26), and binarization was performed (Figure 6a,b). The binarization threshold was designated automatically as the local minimum of the histogram. The morphological opening was the next operation (Figure 6c), which enabled the removal of small objects and smoothed the edges. The interconnected pores were then separated by finding a necking (Figure 6d), and the pores cut with the image frame were deleted (Figure 6e). The analysis of the resulting images (Figure 6f,g) consisted of calculating the parameters concerning the pores.

The same analysis methodology was used for all test samples.

### 2.3. Analysis of Microscopic Images

In order to verify the obtained results of the analysis, measurements were also carried out using the LEXT OLS4000 confocal laser scanning microscope (Olympus, Tokyo, Japan). To obtain confocal micrographs, 10 areas of the test samples were selected for which image acquisition was performed. The analyzed areas were 5120 μm × 5120 μm × 5120 μm.

**Figure 7:**
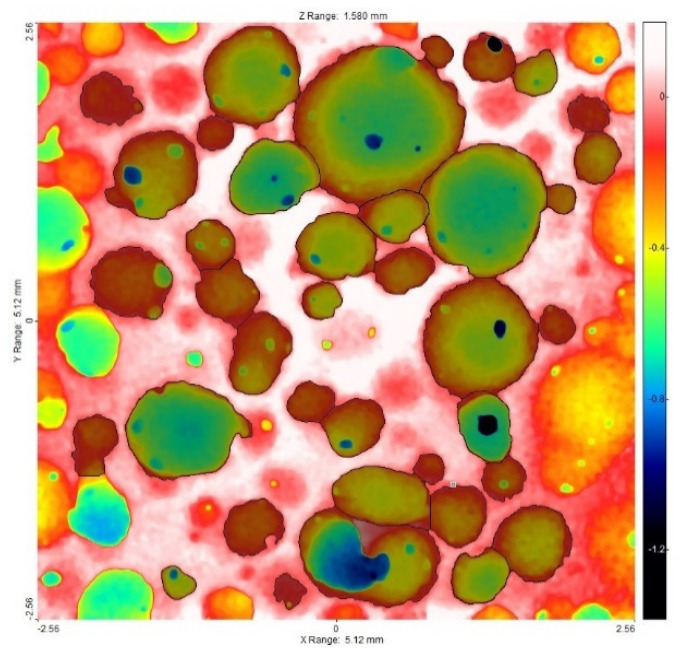
Example of an image resulting from the analysis with SPIP software.

The biomaterial surface images were then analyzed using the SPIP software (version 6.7.9, Image Metrology, Hesholm, Denmark), which enabled the distinction of the surface pores and determined the parameters characterizing their dimensions (Figure 7).

## 3. Results and Discussion

**Figure 8:**
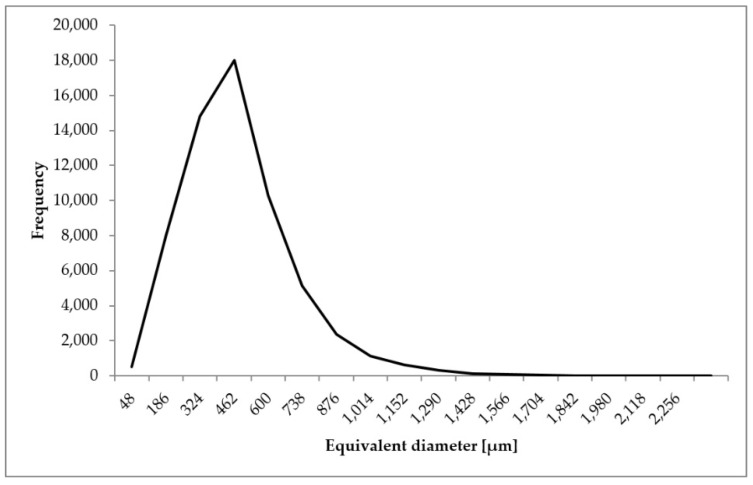
Distribution of equivalent diameter of the analyzed porous biomaterial samples.

**Figure 9:**
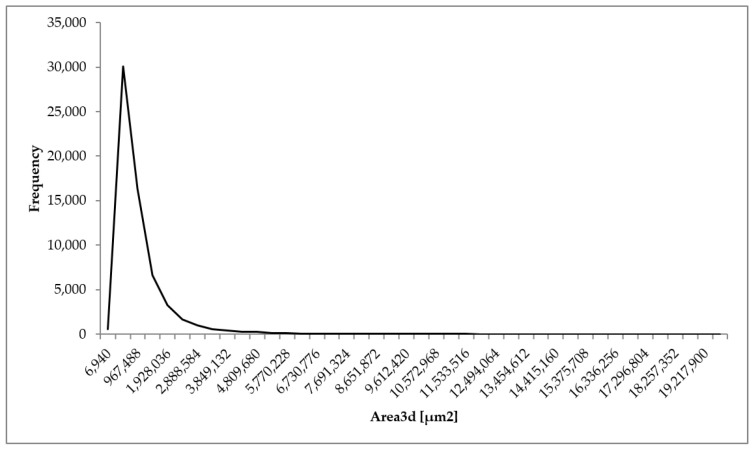
Distribution of 3D area of the analyzed porous biomaterial samples.

**Figure 10:**
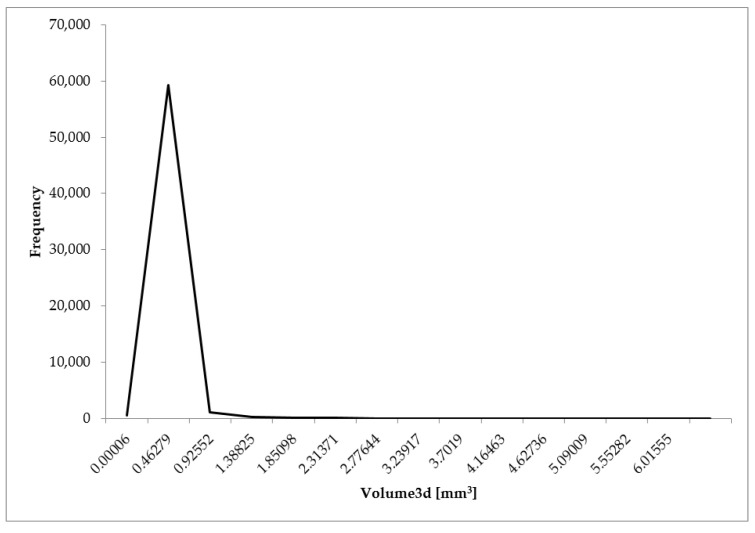
Distribution of 3D volume of the analyzed porous biomaterial samples.

The analysis of the microtomographic images consisted of designating the parameters that characterize the geometric dimensions of the pores and sample porosities. For each of the biomaterial samples, minimum, mean, and maximum values of parameters such as the equivalent diameter, 3D area, and volume were determined. The total volume of all the segmented pores and individual sample porosities expressed as a percentage (the ratio of the pore volume to the total sample volume) were also determined (Table 1, Figure 8, Figure 9 and Figure 10).

The minimum equivalent diameter for all the samples was 48 µm. The mean equivalent diameter ranged from 395 (sample 1) to 427 µm (sample 2). The mean value of the parameter was 411 µm. The maximum equivalent diameter ranged from 2074 for sample 2 to 2256 µm for sample 3. The mean value of the parameter was 2162 µm.

The minimum 3D area of a single pore for all the samples was 6941 µm^2^. The mean 3D area ranged from 689,581 (sample 1) to 920,971 µm^2^ (sample 3). The mean value of the parameter was 787,599 µm^2^. The maximum 3D area ranged from 14,978,800 (sample 2) to 19,217,900 µm^2^ (sample 1). The mean value of the parameter for the samples was 17,235,567 µm^2^.

The minimum volume was 0.00006 mm^3^ for all the samples. The mean volume ranged from 0.05901 (sample 1) to 0.12328 mm^3^ (sample 3). It follows that the value of the mean pore volume for sample 3 is more than twice as high as that for sample 1. The mean value of the parameter was 0.08446 mm^3^. The maximum pore volume ranged from 4.67266 for sample 2 to 6.01555 mm^3^ for sample 3. The mean value of the parameter was 5.31223 mm^3^.

The lowest value of the total pore volume was 1442 mm^3^ for sample 2. Sample 1 (1706 mm^3^) was characterized by the highest value. The mean value of the parameter for the samples was 1552 mm^3^.

The sample porosities are similar. The highest value of porosity was 36% (sample 1) and the lowest 30% (sample 2). The mean porosity was 32%. Samples differ in porosity because, during the manufacturing process, the repeatability of manual mixing and pouring of the slurry into the molds is not ideal. The resulting porous structure is also influenced by the external conditions of the manufacturing process, including the ambient temperature and humidity during molding.

The results obtained during the image analysis indicate the slight geometrical variation of the samples. However, sample 1 has the highest porosity and total pore volume. It is also characterized by the smallest mean diameter, mean 3D area, and mean volume. Moreover, the pores of sample 1 have the largest maximum 3D area of all the test samples.

Sample 2, on the other hand, is characterized by the lowest porosity and total pore volume, as well as the highest mean diameter and pore volume. Moreover, sample 2 has the smallest values of the maximum diameter, maximum 3D area, and maximum pore volume.

The pores of sample 3 have the largest mean 3D area and the largest value of the maximum diameter and maximum pore volume.

**Figure 11:**
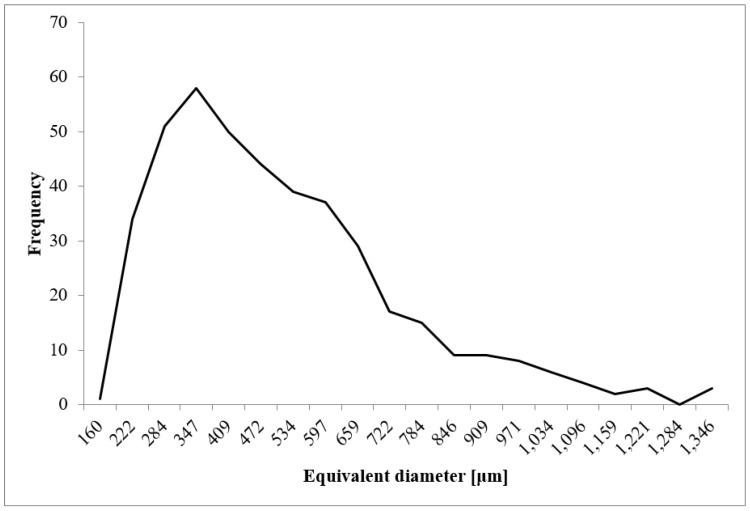
Distribution of pore diameters determined in images obtained using a confocal microscope.

As a result of the analysis of microscopic images carried out using the SPIP program, the mean pore diameter was also determined for the individual images (Table 2, Figure 11). The parameter was to verify the correctness of the results obtained as a result of the above analysis.

It can be seen that the distribution of pore diameters is equivalent to a graph of the probability density function in the Rayleigh distribution.

The value of the mean diameter ranges from 407 to 445 µm. The mean value of the parameter is 426 μm and is in the range between the minimum and maximum values (395–427 µm) obtained during microtomographic examinations of the biomaterial. It can be observed that the results obtained in both stages of the experiment are similar. Therefore, it is concluded that the method determines the correct geometric dimensions of pores, which confirms the correctness of the developed method of analysis.

Previous methods aimed at characterizing the internal structure of porous materials with the use of microtomography were related to specific materials [17,18,19,20,21] and their applications [22] (the studies used microtomography to characterize porosity in terms of material permeability). The analysis proposed in the above articles is similar to the developed method in this study. However, it enables not only determining the porosities of samples but also calculating the parameters characterizing the geometric dimensions of pores, such as the equivalent diameter, 3D area, and pore volume. Therefore, the method can be used in a variety of different applications.

The method enables visualization of the internal structure of porous materials, unlike microscopic methods which do not allow for 3D observations. The sample after the test is suitable for further use because the method is not invasive and does not damage the material during testing, unlike the mercury porosity method in which the mercury remains in the sample, and a high mercury pressure may entail sample distortion. Moreover, the method is simple and requires no special material preparation for testing. The method is also universal, so it can be used for various materials used in implantology.

The determination of the parameters characterizing the geometric dimensions of biomaterial pores is necessary for further stages of research in order to verify the implemented biomaterial pore model. It is a tool for determining the pore volume. The modelling method was tested by comparing the results obtained using the model with the experimental data obtained from a microtomography device [24].

## 4. Conclusions

The main aim of the study was to analyze microtomographic data to determine the geometric dimensions of biomaterial pores.

Samples of a porous corundum biomaterial were studied. The samples were prepared by chemical foaming. In order to obtain 3D images of the samples, measurements were made with an X-ray scanner. Next, 3D images of the samples were generated. Then, an image processing and analysis method was developed using Thermo Scientific Avizo software. The last part of the research involved verifying the developed method by comparing the obtained results with the parameters obtained as a result of microscopic examinations of the biomaterial. The comparison of the results confirmed the correctness of the method.

The developed methodology can be used to analyze biomaterial samples, and it is a tool for determining the geometric dimensions of pores inside a material. The method can be used during the fabrication of a material with a specific pore structure, which will be characterized by a particular volume of pores. Consequently, the determined volume will enable the calculation and then placing of a specific volume of the drug in the open pores of the biomaterial and delivery to the patient’s body during implantation.

## Figures and Tables

**Table 1:** Values of the morphological parameter (equivalent diameter) of the analyzed porous biomaterial samples.

Parameter		Sample 1	Sample 2	Sample 3	Min.	Mean	Max.	Std. Dev.
Equivalent diameter (µm)	min	48	48	48	48	48	48	0
	avg	395	427	410	395	411	427	16.01
	max	2156	2074	2256	2074	2162	2256	91.15
Area3D (µm^2^)	min	6941	6941	6941	6941	6941	6941	0
	avg	689,581	752,246	920,971	689,581	787,599	920,971	119,678
	max	19,217,900	14,978,800	17,510,000	14,978,800	17,235,567	19,217,900	2,132,833
Volume (mm^3^)	min	0.00006	0.00006	0.00006	0.00006	0.00006	0.00006	0
	avg	0.05901	0.07110	0.12328	0.05901	0.08446	0.12328	0.03416
	max	5.24848	4.67266	6.01555	4.67266	5.31223	6.01555	0.67371
	total	1706	1442	1509	1442	1552	1706	137.23
Porosity (%)		36	30	31	30	32	36	3.21

**Table 2:** Parameters determined for individual images obtained using the confocal microscope.

Image/Sample	IM-1/S1	IM-2/S1	IM-3/S1	IM-4/S2	IM-5/S2	IM-6/S2	IM-7/S2	IM-8/S3	IM-9/S3	IM-10/S3	Minimum	Mean	Maximum
Average diameter (μm)	429	445	407	432	421	419	436	414	427	432	407	426	445

## Data Availability

Data Sharing is not applicable.
